# Dual-task gait and white matter hyperintensities in Lewy body diseases: An exploratory analysis

**DOI:** 10.3389/fnagi.2023.1088050

**Published:** 2023-04-05

**Authors:** Ipinuoluwakiye Fatokun, Myrlene Gee, Krista Nelles, Fang Ba, Mahsa Dadar, Simon Duchesne, Breni Sharma, Mario Masellis, Sandra E. Black, Quincy J. Almeida, Eric E. Smith, Frederico Pieruccini-Faria, Manuel Montero-Odasso, Richard Camicioli

**Affiliations:** ^1^Department of Medicine, Division of Neurology, University of Alberta, Edmonton, AB, Canada; ^2^Neuroscience and Mental Health Institute (NMHI), University of Alberta, Edmonton, AB, Canada; ^3^Department of Psychiatry, Douglas Mental Health University Institute, McGill University, Montréal, QC, Canada; ^4^Department of Radiology and Nuclear Medicine, Faculty of Medicine, Laval University, Québec City, QC, Canada; ^5^CERVO Brain Research Centre, Québec City, QC, Canada; ^6^Cumming School of Medicine, University of Calgary, Calgary, AB, Canada; ^7^Hotchkiss Brain Institute, University of Calgary, Calgary, AB, Canada; ^8^Division of Neurology, Department of Medicine, University of Toronto, Toronto, ON, Canada; ^9^Hurvitz Brain Sciences Program, Sunnybrook Research Institute, University of Toronto, Toronto, ON, Canada; ^10^Movement Disorders Research and Rehabilitation Consortium, Department of Kinesiology and Physical Education, Wilfrid Laurier University, Waterloo, ON, Canada; ^11^Department of Clinical Neurosciences, University of Calgary, Calgary, AB, Canada; ^12^Seaman Family MR Research Centre, University of Calgary, Calgary, AB, Canada; ^13^Gait and Brain Lab, Parkwood Institute Research and Lawson Health Research Institute, London, ON, Canada; ^14^Division of Geriatric Medicine, Department of Medicine, Schulich School of Medicine and Dentistry, London, ON, Canada; ^15^Department of Epidemiology and Biostatistics, Schulich School of Medicine and Dentistry, University of Western Ontario, London, ON, Canada

**Keywords:** dual task, gait, white matter hyperintensities, cognition, Lewy body disease, Parkinson’s disease

## Abstract

**Background:**

Parkinson’s disease (PD) and dementia with Lewy bodies (DLB) are part of a spectrum of Lewy body disorders, who exhibit a range of cognitive and gait impairments. Cognitive-motor interactions can be examined by performing a cognitive task while walking and quantified by a dual task cost (DTC). White matter hyperintensities (WMH) on magnetic resonance imaging have also been associated with both gait and cognition. Our goal was to examine the relationship between DTC and WMH in the Lewy body spectrum, hypothesizing DTC would be associated with increased WMH volume.

**Methods:**

Seventy-eight participants with PD, PD with mild cognitive impairment (PD-MCI), PD with dementia or DLB (PDD/DLB), and 20 cognitively unimpaired participants were examined in a multi-site study. Gait was measured on an electronic walkway during usual gait, counting backward, animal fluency, and subtracting sevens. WMH were quantified from magnetic resonance imaging using an automated pipeline and visual rating. A median split based on DTC was performed. Models included age as well as measures of global cognition and cardiovascular risk.

**Results:**

Compared to cognitively unimpaired participants, usual gait speed was lower and DTC was higher in PD-MCI and PDD/DLB. Low DTC participants had higher usual gait speed. WMH burden was greater in high counting DTC participants. Frontal WMH burden remained significant after adjusting for age, cardiovascular risk and global cognition.

**Conclusion:**

Increased DTC was associated with higher frontal WMH burden in Lewy body disorders after adjusting for age, cardiovascular risk, and global cognition. Higher DTC was associated with age.

## Introduction

Lewy body disorders (LBD) represent a spectrum of disorders, characterized by the over-accumulation of alpha-synuclein in the brain, leading to the formation of Lewy bodies, which are associated with neuronal loss (Galasko, 2017; Kouli et al., 2018). Neuronal loss progression in subcortical and cortical brain regions results in increasing motor (including gait) and cognitive impairment (Galasko, 2017; Mirelman et al., 2019, 2021). Consequently, diagnoses range from Parkinson’s disease (PD) with intact cognition, PD with mild cognitive impairment (PD-MCI) to PD with dementia (PDD) and dementia with Lewy bodies (DLB) (grouped as PDD/DLB).

Gait slowing in aging, especially while performing a simultaneous cognitive task (dual task), is thought to be an early predictor of significant cognitive decline (Mielke et al., 2012; Montero-Odasso et al., 2012a). The extent of slowing can be quantified by dual task cost (DTC), which indicated slowing during a secondary task, relative to the participant’s usual task gait speed (Montero-Odasso et al., 2012b). Essentially, DTC reflects cognitive-motor reserve. While it is clear that gait is affected by dual tasks in healthy older adults (Smith et al., 2017), patients with LBD (Raffegeau et al., 2019), and older people with cognitive decline (Montero-Odasso et al., 2017) associated brain imaging correlates are not well established (Veldkamp et al., 2021).

White matter lesions presenting as white matter hyperintensities (WMH) on magnetic resonance imaging (MRI) have also been related to cognitive and gait deficits in healthy aging (Crockett et al., 2022), cerebral small vessel disease (Sharma et al., 2022), and PD (Toda et al., 2019; Dadar et al., 2020). Some studies have found participants with PDD may have greater WMH volumes than controls and PD with intact cognition (Butt et al., 2021).

Overall, the relationship between dual task gait change and WMH in the Lewy body spectrum is not well understood. To address this gap, we explored the association between white matter changes and DTC across the Lewy body spectrum. We hypothesized that WMH would be associated with DTC, regardless of the secondary task.

## Materials and methods

Participants were enrolled in the multi-center Canadian Consortium on Neurodegeneration in Aging’s (CCNA), Comprehensive Assessment of Neurodegeneration and Dementia (COMPASS-ND) study (Chertkow et al., 2019) and the Functional Assessment and Vascular Reactivity (FAVR)-II study (Beaudin et al., 2022). The COMPASS-ND was approved by the research ethics boards of all the involved institutions while FAVR-II was approved at the University of Alberta and University of Calgary. Both studies were carried out in accordance with the Code of Ethics of the World Medical Association. Participants provided their written informed consent to participate.

### Participants

Participants were recruited from movement disorder and cognitive clinics as well as referrals from community physicians and community advertisements. Three COMPASS-ND study sites (University of Alberta, University of Calgary, and the Sunnybrook Research Institute in Toronto) completed assessments with electronic walkways. Cognitively unimpaired (CU) participants from FAVR-II, which is harmonized with COMPASS-ND, were recruited at the University of Alberta and University of Calgary. Sequential male and female participants from the recruiting sites were included. All met published criteria for a LBD or were CU as outlined previously (Chertkow et al., 2019; Pieruccini-Faria et al., 2021). Patients with severe cognitive impairment (MoCA <13), active neuro-psychiatric problems or immobility were not included.

Eighty-two participants diagnosed with PD or DLB were included from the COMPASS-ND cohort: 42 had PD without cognitive impairment (PD), 20 had PD-MCI, 9 had PDD, and 11 were diagnosed with DLB. Initial diagnosis criteria included Montreal Cognitive Assessment (MoCA) score (range 0–30, higher score represents better cognition) where a score ≤24 indicated PD-MCI or PDD/DLB. The PDD/DLB group had sufficient cognitive impairment to interfere with independent function. A MoCA score between 8 and 20 inclusive was considered indicative of dementia. Final diagnosis was based on further evaluation by an experienced neurologist (RC/ES/MM/SEB) using established criteria as previously described (Pieruccini-Faria et al., 2021). Given the small number of participants and that PDD and DLB are both Lewy body dementias with overlapping pathological features (Galasko, 2017), the PDD and DLB groups were combined for statistical analysis (PDD/DLB). CU participants were included, with 13 from COMPASS-ND and 7 from FAVR-II. Four participants were unable to undergo MRI and subsequently excluded. The final number of participants for analysis was 20 CU, 41 PD, 17 PD-MCI, and 20 PDD/DLB.

### Clinical assessment

Demographic descriptors included age, sex, and years of education. Global cognition was measured using MoCA. Cardiovascular health was summarized using the Framingham cardiovascular risk score (D’Agostino et al., 2008), which includes age, sex, diabetes, current smoking status, systolic blood pressure, treatment for hypertension, and body mass index. Patients were characterized by disease duration, levodopa equivalent daily dose (LEDD) (Tomlinson et al., 2010), and Movement Disorder Society-Unified Parkinson’s Disease Rating Scale Part 3 (MDS-UPDRS III) (range 0–132, higher score indicates greater severity) (Goetz et al., 2008). The patients were in the ON state when their gait was tested if they were on dopaminergic medications. They were tested at a time they were comfortable with doing the walking task. While dyskinesia were present in some patients these did not interfere with the walking tasks. Self-reported gait freezing was assessed using the Freezing of Gait Questionnaire (range 0–24, higher score indicates greater severity of gait impairment) (Giladi et al., 2000).

### Gait measurement and analysis

Gait was evaluated according to the standardized COMPASS-ND protocol (Cullen et al., 2018). A ProtoKinetics Zeno Walkway (Edmonton and Calgary) or a GAITRite (Sunnybrook) walkway was used to measure gait parameters from 6 m walks (Cullen et al., 2018). To ensure steady gait speed on the walkway and minimum acceleration and deceleration effects, walks commenced 1 m before and ended 1 m after the gait mat. Usual gait was measured while participants walked at a comfortable pace. Three trials were performed to calculate average usual gait. Participants were then instructed to walk while simultaneously engaging in the following verbal tasks in a fixed order: (1) counting backward by 1s starting from 100 (“counting”), (2) naming as many animals as possible without repetition (“fluency”), and (3) counting backward by 7s starting from 100 (“serial 7s”). Performance on the verbal tasks were measured by the number of correct subtractions for counting and serial 7s, and the number of unique animals named for fluency. The cognitive task performance was recorded but cognitive task costs were not analyzed.

The following formula was used to calculate the DTC on gait speed for each condition (Montero-Odasso et al., 2012b):


$$D\,T\,C=\left(\frac{\left[u\,s\,u\,a\,l\,g\,a\,i\,t\,s\,p\,e\,e\,d-d\,u\,a\,l\,g\,a\,i\,t\,s\,p\,e\,e\,d\right]}{u\,s\,u\,a\,l\,g\,a\,i\,t\,s\,p\,e\,e\,d}\right)\times 100$$

### Evaluation of WMH on MRI

All MRI scans were completed on a Siemens Prisma 3.0 T system (Edmonton and Sunnybrook) or 3.0 T GE Discovery MR750 (Calgary), according to the Canadian Dementia Imaging Protocol (Duchesne et al., 2018). T1-weighted, T2-weighted, and fluid-attenuated inversion recovery images were used to measure WMH volumes with an automated tool (Dadar et al., 2017). Total WMH volume was normalized for intracranial volume and log_10_ transformed to obtain a normal distribution. Additionally, the presence and severity of WMH was rated qualitatively using Fazekas Visual Rating Scale (Fazekas et al., 1987). Illustrative images and technical details are published (Dadar et al., 2022).

### Statistical analysis

Data was analyzed using SPSS (Version 26, IBM Corporation, Armonk, NY, USA). One-way analysis of variance (ANOVA) was used for continuous variables and Chi-squared test for categorical variables. Two analyses were performed, (1) across the groups (CU, PD-MCI, and PDD/DLB) where we looked at overall group comparisons and pair wise comparisons between the groups. The Sidak correction for multiple corrections was used for *post-hoc* pairwise comparisons between the groups, and (2) high vs. low DTC groups within the Lewy body spectrum.

First, clinical characteristics, gait and dual task gait were compared across groups for each task separately using ANOVAs or ANCOVAs. For all three dual task conditions, DTC was not normally distributed and attempts to normalize with commonly used transformations such as log_10_, various functions (powers, exponential, and arcsinh), and a Box Cox transformation failed. Consequently, and based on the observation that some participants show no DTC, while others showed a range of increased cost, a median split of DTC for each task within the Lewy body group, excluding CU, was used to convert DTC into a categorical variable.

Differences in WMH volumes between the high and low DTC groups were modeled to explore contributions from covariates. An ANOVA model was first used to compare log_10_ transformed WMH volumes between high and low DTC groups across the Lewy body spectrum (model 1). Subsequent analysis of covariance (ANCOVA) was performed with age as a covariate (model 2) due to its potential association with gait, cognition, and white matter changes. Cardiovascular risk (FCRS) was added (model 3) due to the established relationship with WMH (Moroni et al., 2018). The impact of global cognition was evaluated by including MoCA (model 4). While MDS-UPRDS III did not differ significantly between the high and low DTC group, it has been shown to correlate with WMH volumes (Chen et al., 2021; Jeong et al., 2021); hence, supplementary modeling was performed with it as a covariate.

Estimated marginal means ± standard deviations are reported for the ANOVAs and ANCOVAs. A threshold of *p* < 0.05 was considered statistically significant. With the exception of multiple pair wise comparisons across study groups, multiple comparisons corrections were not performed, given the exploratory nature of the study.

## Results

### Controls and Lewy body spectrum groups

**FIGURE 1:**
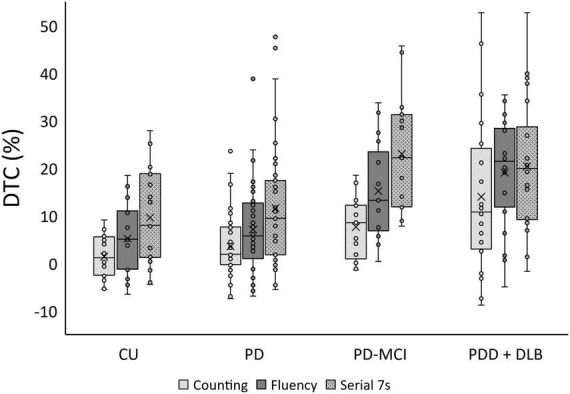
Boxplot of dual task cost (DTC) % for cognitively unimpaired health participants (CU), Parkinson’s disease (PD), Parkinson’s disease with mild cognitive impairment (PD-MCI), and a combined group of participants with either Parkinson’s disease with dementia (PDD) or Dementia with Lewy Bodies (DLB).

Differences in age and sex proportion of the four study groups (CU, PD, PD-MCI, and PDD/DLB) were statistically significant (Table 1). Global cognition, as assessed by MoCA, was significantly different between groups (*p* < 0.001) as expected. Education did not differ significantly between groups (*p* = 0.99). Framingham cardiovascular risk score differed significantly between groups (*p* = 0.01) and was highest in PD-MCI and PDD/DLB groups (*post-hoc* CU vs. PD: *p* = 0.9, CU vs. PD-MCI: *p* = 0.02, CU vs. PDD/DLB: *p* = 0.03). The groups did not differ significantly with respect to disease duration (*p* = 0.4) or MDS-UPDRS III (*p* = 0.1). LEDD significantly differed between the groups (*p* = 0.04); PD-MCI had higher LEDD than PDD/ DLB (*post-hoc p* = 0.03). Self-reported gait freezing differed between the PD groups (*p* = 0.03) with the PDD/ DLB group reporting greater freezing than PD (*post-hoc p* = 0.009) while the PD and PD-MCI groups were similar (*post-hoc p* = 0.2). Baseline gait speed significantly differed between groups (*p* < 0.001) with both the PD-MCI and PDD/DLB groups being slower than the CU and PD groups (*post-hoc* CU vs. PD-MCI: *p* = 0.004, CU vs. PDD/DLB: *p* < 0.001, CU vs. PD, *p* = 0.2). The DTC for counting, fluency, and serial 7s were all significantly different between groups (*p* < 0.001 for counting and fluency, *p* = 0.001 for serial 7s). For all tasks, PD-MCI and PDD/DLB participants had significantly higher DTC compared to CU (*post-hoc* CU vs. PD-MCI: *p* = 0.04 for counting, *p* = 0.002 for fluency and serial 7s; CU vs. PDD/DLB: *p* < 0.001 for counting and fluency, *p* = 0.007 for serial 7s). Cognitive task performance was similar in all groups for counting (*p* = 0.2); but differed for fluency (*p* < 0.001), where the PDD/DLB group performed worse than CU (*post-hoc p* = 0.02), and for serial 7s (*post-hoc p* = 0.001), where the PD group performed better than the other groups (*post-hoc* PD vs. CU: *p* = 0.05, PD vs. PD-MCI: *p* = 0.01, PD vs. PDD/DLB: *p* < 0.001). We show DTC by group, including the control group in Figure 1.

**TABLE 1:** Demographics and DTC variables for all groups.

	CU	PD	PD-MCI	PDD/DLB	*p*
*N*	20	41	17	20	–
Age (years)	68.7 ± 5.8	66.7 ± 7.2	70.5 ± 8.0	72.9 ± 8.5	**0.02**
Females, *N* (%)	15 (75.0)	22 (53.7)	3 (17.6)	2 (10.0)	<**0.001**
Education (years)	16.1 ± 2.9	15.8 ± 3.0	15.6 ± 3.3	16.2 ± 5.0	1
FCRS^†^ (%)	18.9 ± 14.6	19.3 ± 14.4	30.0 ± 11.7	28.7 ± 14.0	**0.01**
MoCA	27.5 ± 1.8	27.9 ± 1.4	22.4 ± 4.3	18.70 ± 4.4	<**0.001**
Disease duration (years)	–	6.4 ± 3.7	8.0 ± 5.9	7.8 ± 5.8	0.4
MDS-UPDRS III	–	21.2 ± 10.7	26.2 ± 12.5	29.0 ± 19.0	0.1
LED (mg)	–	640 ± 375	852 ± 477	469 ± 531	0.1
FOG-Q	–	3.4 ± 3.6	5.1 ± 5.4	7.0 ± 6.3	**0.03**
Baseline gait speed (cm/s)	141.1 ± 16.2	132.6 ± 23.9	119.3 ± 22.5	104.5 ± 23.1	<**0.001**
**Counting**					
DTC (%)	1.4 ± 4.5	3.5 ± 6.6	7.6 ± 6.3	14.0 ± 16.8	<**0.001**
Correct subtractions	6.8 ± 1.8	8.3 ± 2.3	8.4 ± 2.3	7.8 ± 3.9	0.2
**Fluency**					
DTC (%)	5.2 ± 7.3	7.1 ± 9.0	15.2 ± 9.8	19.1 ± 11.4	<**0.001**
Number named	6.0 ± 1.0	6.6 ± 1.6	5.6 ± 1.3	4.8 ± 1.7	<**0.001**
**Serial 7s**					
DTC (%)	9.6 ± 10.1	11.6 ± 12.5	23.0 ± 12.1	20.4 ± 14.5	**0.001**
Correct subtractions	2.8 ± 1.7	3.8 ± 1.9	2.5 ± 1.6	2.0 ± 1.9	**0.001**
Raw WMH volume (mm^3^)*					
Total	6,210 ± 4,833	8,790 ± 11,543	14,541 ± 15,566	20,581 ± 16,742	**0.001**
Frontal lobe	3,207 ± 2,757	43,89 ± 5,842	7,051 ± 7,397	8,912 ± 6,562	**0.008**
Temporal lobe	730 ± 575	968 ± 990	1,575 ± 1,417	2,341 ± 2,607	**0.002**
Parietal lobe	1,406 ± 1,440	2,315 ± 4,409	4,234 ± 5,949	6,573 ± 6,821	**0.004**
Occipital lobe	864 ± 596	1,111 ± 1,103	1,658 ± 1,271	2,747 ± 2,026	**< 0.001**
**Fazekas score^#^**					
Total	1.50 ± 1.10	2.22 ± 1.30	2.65 ± 1.27	2.90 ± 1.21	**0.003**
Periventricular	0.60 ± 0.60	1.10 ± 0.70	1.35 ± 0.70	1.80 ± 0.83	**<0.001**
Subcortical	0.90 ± 0.72	1.12 ± 78	1.29 ± 0.69	1.10 ± 0.55	0.4

PD, Parkinson’s disease; PD-MCI, Parkinson’s disease with mild cognitive impairment; PDD, Parkinson’s disease with dementia; DLB, dementia with Lewy bodies; FCRS, Framingham cardiovascular risk score; MoCA, Montreal Cognitive Assessment; MDS-UPDRS III, Movement Disorders Society-Unified Parkinson’s Disease Rating Scale; FOG-Q, Freezing of Gait Questionnaire; LED, levodopa equivalent dose; DTC, dual task cost; WMH, white matter hyperintensities. ^†^Missing for one participant. *Not significant after for correction for age and sex, except for occipital WMH. p-Values for raw comparisons are shown. ^#^Significant for total and periventricular scores after correction for age and sex. p-Values for raw comparisons are shown. Bold values represent the statistically significant.

**FIGURE 2:**
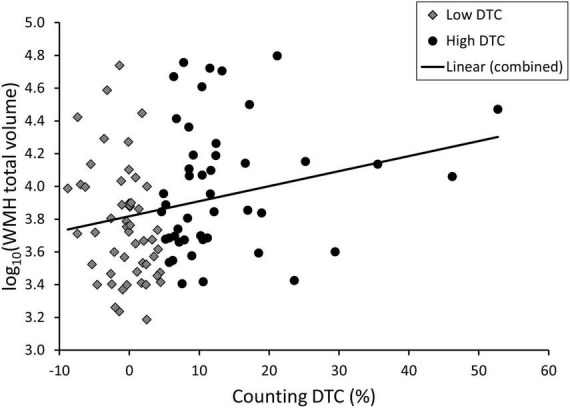
Scatterplot of total white matter hyperintensity (WMH) volume and dual task cost (DTC) under the counting condition for patients with Lewy body disorders with high vs. low DTC (median split).

**FIGURE 3:**
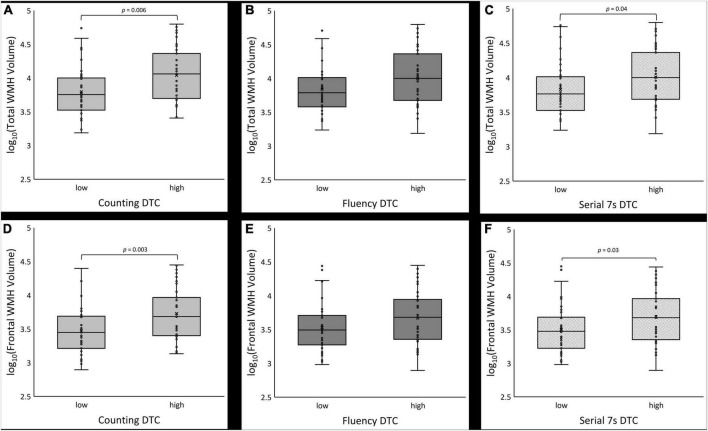
Boxplots of log_10_-transformed total white matter hyperintensity (WMH) volume **(A–C)** and frontal lobe WMH volume **(D–F)** for low vs. high dual task cost (DTC) under counting, fluency and serial 7s conditions, respectively.

White matter hyperintensities volume was significantly different across groups for total volume (*p* = 0.001) as well as each lobe (frontal: *p* = 0.008, temporal: *p* = 0.002, parietal, *p* = 0.004, occipital: *p* < 0.001), though this was not significant after correction for age and sex except for the occipital lobe. In uncorrected post hoc comparisons, the PDD/DLB group was significantly different from CU and PD but not PD-MCI with respect to total WMH volume (*post-hoc* CU vs. PDD/DLB: *p* = 0.003, PD vs. PDD/DLB: *p* = 0.005) as well as all lobes (*post-hoc* CU vs. PDD/DLB: frontal *p* = 0.02, temporal *p* = 0.005, parietal *p* = 0.001, occipital *p* < 0.001; PD vs. PDD/DLB: frontal *p* = 0.03, temporal *p* = 0.006, parietal *p* = 0.002, occipital *p* < 0.001). The differences in WMH burden were also evident qualitatively *via* Fazekas score, where total score (*p* = 0.003) and periventricular score (*p* < 0.001) were significantly different between groups, and remained significant after correction for age and sex; the total Fazekas score was greater in PD-MCI and PDD/DLB compared to CU (*post-hoc* CU vs. PD-MCI: *p* = 0.03, CU vs. PDD/DLB: *p* = 0.003) and the periventricular score was greater in all three PD groups vs. CU (*post-hoc* CU vs. PD: *p* = 0.04, CU vs. PD-MCI: *p* = 0.005, CU vs. PDD/DLB: *p* = 0.001). In addition, the PDD/DLB had a higher periventricular score than the PD group (*post-hoc p* = 0.001). We show the association between DTC and WMH in Figure 2. We show the difference in total and frontal WMH for each dual task divided by median split in Figure 3.

### High vs. low DTC groups

The DTC medians for the Lewy body spectrum were 4.5% for counting, 10.9% for fluency, and 14.5% for serial 7s. After median splitting the Lewy body spectrum participants by DTC, the difference in age was statistically significant in all dual task conditions (Table 2), with the low DTC group being younger (counting: *p* = 0.04, fluency: *p* = 0.03, serial 7s: *p* = 0.03). Sex, years of education, Framingham cardiovascular risk score, disease duration, and LEDD did not significantly differ between the low and high DTC groups across conditions. For all conditions, MoCA was significantly different, with the low DTC group performing better than the high DTC group (counting: *p* = 0.01, fluency: *p* < 0.001, serial 7s: *p* = 0.001). The proportion of patients with freezing of gait only differed between the high and low serial 7s DTC groups (*p* = 0.008), with the high DTC group reporting significantly greater gait freezing. A higher proportion of PD participants were in the low DTC groups, and the majority of PD-MCI and PDD/DLB participants were in the high DTC groups for all conditions (counting: *p* = 0.01, fluency: *p* < 0.001, serial 7s: *p* = 0.01).

**TABLE 2:** Demographics and dual task cost (DTC) variables for median split data without cognitively unimpaired healthy participants.

	Low DTC	High DTC	*p*
**Counting DTC**			
Age (years)	68.2 ± 8.2	71.0 ± 7.6	**0.04**
Females, *N* (%)	13 (33.3)	14 (35.9)	0.8
Education (years)	15.9 ± 2.9	15.9 ± 4.3	1
FCRS (%)	25.6 ± 16.7	22.4 ± 11.9	0.3
MoCA	25.8 ± 4.0	22.9 ± 5.6	**0.01**
Disease duration (years)	6.9 ± 4.4	7.3 ± 5.2	0.7
MDS-UPDRS III	23.1 ± 13.7	25.4 ± 14.1	0.5
LED (mg)	580 ± 366	706 ± 527	0.2
FOG-Q	4.6 ± 5.0	4.7 ± 5.0	0.9
Baseline gait speed (cm/s)	130.5 ± 21.4	114.51 ± 27.88	**0.006**
DTC (%)	−0.5 ± 3.5	14.7 ± 10.6	<**0.001**
Correct on cognitive task	8.6 ± 2.0	8.0 ± 3.4	0.3
**Raw WMH volume (mm^3^)**			
Total	9,261 ± 10,779	16,873 ± 16,939	**0.02**
Frontal lobe	4,279 ± 4,898	7,978 ± 7,564	**0.01**
Temporal lobe	1,144 ± 1,050	1,760 ± 2,157	0.1
Parietal lobe	2,414 ± 3,776	5,236 ± 6,840	**0.03**
Occipital lobe	1,412 ± 1,630	1,887 ± 1,478	0.2
Fazekas score			
Total	2.2 ± 1.1	2.8 ± 1.4	**0.03**
Periventricular	1.1 ± 0.6	1.5 ± 0.8	**0.01**
Subcortical	1.1 ± 0.6	1.3 ± 0.8	0.2
PD**/**PD-MCI**/**PDD**/**DLB (%)	70/15/15	36/28/36	**0.01**
**Fluency DTC**			
Age (years)	67.1 ± 7.5	71.1 ± 8.2	**0.03**
Females, *N* (%)	15 (38.5)	12 (30.8)	0.5
Education (years)	16.0 ± 2.6	15.8 ± 4.5	0.8
FCRS (%)	22.6 ± 15.2	25.4 ± 13.7	0.4
MoCA	26.2 ± 4.1	22.4 ± 5.2	<**0.001**
Duration of disease (years)	7.3 ± 4.9	7.0 ± 4.8	0.8
MDS-UPDRS III	22.2 ± 3.2	26.3 ± 14.4	0.2
LED (mg)	656 ± 442	630 ± 471	0.8
FOG-Q	4.5 ± 4.7	4.9 ± 5.3	0.7
Baseline gait speed (cm/s)	131.9 ± 22.5	113.1 ± 26.1	**0.001**
DTC (%)	3.0 ± 4.8	20.9 ± 7.8	<**0.001**
Correct on cognitive task	6.1 ± 1.7	5.8 ± 1.8	0.5
**Raw WMH volume (mm^3^)**			
Total	103,712 ± 12,160	15,762 ± 16,434	0.1
Frontal lobe	5,130 ± 6,060	7,127 ± 7,066	0.2
Temporal lobe	1,100 ± 1,003	1,803 ± 2,166	0.07
Parietal lobe	2,769 ± 4,603	4,881 ± 6,455	1.0
Occipital lobe	1,364 ± 1,489	1,934 ± 1,605	1.0
**Fazekas score**			
Total	2.3 ± 1.3	2.6 ± 1.3	0.3
Periventricular	1.2 ± 0.6	1.5 ± 0.8	0.06
Subcortical	1.2 ± 0.8	1.2 ± 0.6	1
PD**/**PD-MCI/PDD**/**DLB (%)	74/13/13	31/31/38	<**0.001**
**Serial 7s DTC**			
Age (years)	67.7 ± 7.2	70.5 ± 8.8	**0.03**
Females, *N* (%)	15 (38.5)	12 (30.8)	0.5
Education (years)	16.0 ± 3.8	15.7 ± 3.4	0.7
FCRS (%)	23.6 ± 16.0	24.3 ± 12.9	0.8
MoCA	26.1 ± 4.1	22.5 ± 5.3	**0.001**
Duration of disease (years)	6.2 ± 3.8	8.0 ± 5.5	0.09
MDS-UPDRS III	22.5 ± 12.5	26.0 ± 15.1	0.3
LED (mg)	560 ± 391	686 ± 514	0.4
FOG-Q	3.2 ± 3.6	6.2 ± 5.7	**0.008**
Baseline gait speed (cm/s)	132.2 ± 20.0	112.8 ± 27.9	<**0.001**
DTC (%)	5.4 ± 5.4	27.2 ± 10.6	<**0.001**
Correct on cognitive task	3.8 ± 2.1	2.32 ± 1.7	<**0.001**
**Raw WMH volume (mm^3^)**			
Total	1,0434 ± 2,951	15,700 ± 5,840	0.1
Frontal lobe	4,830 ± 5,946	7,427 ± 7,029	0.08
Temporal lobe	1,203 ± 1,269	1,701 ± 2,053	0.2
Parietal lobe	2,868 ± 4,699	4,783 ± 6,416	0.1
Occipital lobe	1,516 ± 1,658	1,780 ± 1,476	0.5
Fazekas score			
Total	2.3 ± 1.2	2.7 ± 1.4	0.2
Periventricular	1.2 ± 0.6	1.4 ± 0.8	0.2
Subcortical	1.1 ± 0.7	1.2 ± 0.7	0.3
PD**/**PD-MCI**/**PDD/DLB (%)	70/15/15	36/28/36	**0.01**

DTC, dual task cost; FCRS, framingham cardiovascular risk score; MoCA, Montreal Cognitive Assessment; MDS-UPDRS III, Movement Disorders Society-Unified Parkinson’s Disease Rating Scale; LED, levodopa equivalent dose; FOG-Q, Freezing of Gait Questionnaire; WMH, white matter hyperintensities; PD, Parkinson’s disease; PD-MCI, Parkinson’s disease with mild cognitive impairment; PDD, Parkinson’s disease with dementia; DLB, dementia with Lewy bodies. Bold values represent the statistically significant.

As expected, mean DTC and baseline gait speed differed significantly between the low and high DTC groups for all conditions (counting: *p* = 0.006, fluency: *p* = 0.001, serial 7s: *p* < 0.001). Mean DTC for the low DTC groups was significantly lower than the mean for the high group (*p* < 0.001 for all conditions). For usual gait speed, the low DTC group was consistently faster than the high DTC group (counting: *p* = 0.006, fluency: *p* = 0.001, serial 7s: *p* < 0.001). Performance on the cognitive task did not differ between high and low DTC groups for counting (*p* = 0.3) or fluency (*p* = 0.5); however, for serial 7s, the low DTC had more correct subtractions than the high DTC group (*p* < 0.001). Total WMH volume was significantly different between high and low DTC groups for counting only (*p* = 0.02), with the differences predominantly in the frontal (*p* = 0.01) and parietal (*p* = 0.03) lobes. For fluency and serial 7s, the differences in total and lobar WMH volumes were not significantly different between the high and low DTC groups. Similar trends were observed with Fazekas scoring, where the high and low DTC groups for counting differed in total score (*p* = 0.03) and periventricular score (*p* = 0.01), but not for fluency or serial 7s.

#### Counting dual task cost

Boxplots of log_10_ transformed WMH volumes are shown in Figures 3A, D for the counting condition. The low DTC group had lower WMH burden which was statistically significant in three of the four models (Table 3). Without covariates (model 1), the estimated difference in the log_10_ transformed total WMH volume between the low vs. high DTC group was 0.245 (*p* = 0.006). The frontal, parietal, and occipital lobes had greater WMH burden in the high DTC group compared to the low DTC group (difference = 0.259, *p* = 0.003 for frontal, difference = 0.319, *p* = 0.008 for parietal, and difference = 0.177, *p* = 0.04 for occipital).

**TABLE 3:** Models of linear univariate analysis with estimated marginal means.

Condition	Lobe	Log_10_ (WMH volume)		Estimated difference	*p*-value	Adjusted *R*^2^
		Low DTC	High DTC			
**MODEL # 1: no covariates**						
Counting DTC	Total	3.80 ± 0.38	4.04 ± 0.38	0.245	**0.006**	0.083
	Frontal	3.47 ± 0.37	3.73 ± 0.37	0.259	**0.003**	0.100
	Temporal	2.90 ± 0.39	3.04 ± 0.39	0.143	0.1	0.02
	Parietal	3.08 ± 0.52	3.40 ± 0.52	0.319	**0.008**	0.078
	Occipital	2.97 ± 0.37	3.15 ± 0.37	0.177	**0.04**	0.044
Fluency DTC	Total	3.84 ± 0.39	4.00 ± 0.39	0.156	0.08	0.026
	Frontal	3.53 ± 0.39	3.67 ± 0.39	0.138	0.1	0.019
	Temporal	2.89 ± 0.39	3.05 ± 0.39	0.154	0.09	0.025
	Parietal	3.13 ± 0.53	3.35 ± 0.53	0.222	0.07	0.031
	Occipital	2.97 ± 0.37	3.15 ± 0.37	0.177	**0.04**	0.043
Serial 7s DTC	Total	3.83 ± 0.39	4.01 ± 0.39	0.181	**0.04**	0.040
	Frontal	3.51 ± 0.38	3.69 ± 0.38	0.180	**0.03**	0.054
	Temporal	2.89 ± 0.39	3.04 ± 0.39	0.150	0.1	0.023
	Parietal	3.12 ± 0.52	3.35 ± 0.52	0.230	0.06	0.034
	Occipital	2.97 ± 0.37	3.15 ± 0.37	0.177	**0.04**	0.043
**MODEL #2: age**						
Counting DTC	Total	3.85 ± 0.31	3.98 ± 0.31	0.133	0.07	0.413
	Frontal	3.52 ± 0.31	3.68 ± 0.31	0.154	**0.03**	0.409
	Temporal	2.95 ± 0.34	2.99 ± 0.34	0.040	0.6	0.313
	Parietal	3.15 ± 0.42	3.32 ± 0.42	0.169	0.08	0.406
	Occipital	3.01 ± 0.33	3.11 ± 0.33	0.100	0.2	0.253
Fluency DTC	Total	3.90 ± 0.32	3.93 ± 0.32	0.035	0.6	0.388
	Frontal	3.59 ± 0.31	3.61 ± 0.31	0.020	0.8	0.372
	Temporal	2.95 ± 0.34	2.99 ± 0.34	0.045	0.6	0.314
	Parietal	3.21 ± 0.42	3.27 ± 0.42	0.061	0.5	0.384
	Occipital	3.02 ± 0.33	3.11 ± 0.33	0.090	0.2	0.252
Serial 7s DTC	Total	3.87 ± 0.32	3.96 ± 0.32	0.094	0.2	0.400
	Frontal	3.55 ± 0.31	3.65 ± 0.31	0.103	0.1	0.389
	Temporal	2.93 ± 0.33	3.00 ± 0.33	0.072	0.3	0.319
	Parietal	3.18 ± 0.42	3.29 ± 0.42	0.114	0.2	0.392
	Occipital	3.03 ± 0.33	3.09 ± 0.33	0.060	0.5	0.243
**MODEL #3: age and FCRS^†^**						
Counting DTC	Total	3.84 ± 0.31	4.00 ± 0.31	0.163	**0.03**	0.428
	Frontal	3.52 ± 0.31	3.69 ± 0.31	0.174	**0.02**	0.414
	Temporal	2.93 ± 0.33	3.01 ± 0.33	0.080	0.3	0.351
	Parietal	3.13 ± 0.42	3.35 ± 0.42	0.213	**0.03**	0.428
	Occipital	3.00 ± 0.34	3.12 ± 0.33	0.121	0.1	0.266
Fluency DTC	Total	3.90 ± 0.31	3.94 ± 0.32	0.039	0.6	0.391
	Frontal	3.59 ± 0.32	3.62 ± 0.32	0.024	0.7	0.369
	Temporal	2.95 ± 0.33	3.00 ± 0.33	0.049	0.5	0.344
	Parietal	3.21 ± 0.43	3.28 ± 0.42	0.068	0.5	0.395
	Occipital	3.02 ± 0.34	3.11 ± 0.33	0.094	0.2	0.257
Serial 7s DTC	Total	3.88 ± 0.31	3.97 ± 0.31	0.091	0.2	0.401
	Frontal	3.56 ± 0.31	3.65 ± 0.32	0.096	0.2	0.382
	Temporal	2.93 ± 0.33	3.01 ± 0.33	0.078	0.3	0.350
	Parietal	3.19 ± 0.42	3.29 ± 0.43	0.105	0.3	0.400
	Occipital	3.04 ± 0.34	3.09 ± 0.34	0.040	0.5	0.246
**MODEL #4: age, FCRS, and MoCA^†^**						
Counting DTC	Total	3.85 ± 0.32	4.00 ± 0.32	0.150	0.053	0.423
	Frontal	3.52 ± 0.32	3.68 ± 0.32	0.166	**0.03**	0.407
	Temporal	2.93 ± 0.34	3.02 ± 0.34	0.088	0.3	0.342
	Parietal	3.15 ± 0.44	3.33 ± 0.43	0.187	0.07	0.427
	Occipital	3.02 ± 0.34	3.10 ± 0.34	0.080	0.3	0.288
Fluency DTC	Total	3.92 ± 0.33	4.00 ± 0.32	0.012	0.9	0.392
	Frontal	3.60 ± 0.32	3.60 ± 0.33	0.000	1	0.368
	Temporal	2.95 ± 0.34	3.00 ± 0.34	0.050	0.5	0.335
	Parietal	3.23 ± 0.44	3.25 ± 0.44	0.023	0.8	0.401
	Occipital	3.04 ± 0.34	3.08 ± 0.34	0.045	0.6	0.281
Serial 7s DTC	Total	3.89 ± 0.32	3.96 ± 0.33	0.071	0.4	0.399
	Frontal	3.56 ± 0.32	3.64 ± 0.32	0.081	0.3	0.377
	Temporal	2.93 ± 0.34	3.02 ± 0.34	0.083	0.3	0.341
	Parietal	3.21 ± 0.43	3.28 ± 0.44	0.067	0.5	0.404
	Occipital	3.06 ± 0.33	3.06 ± 0.33	0.000	1	0.378

**^†^**One participant was missing data needed to calculate FCRS thus is not included in analysis for Models #3 and 4. WMH, white matter hyperintensity; DTC, dual task cost; FCRS, framingham cardiovascular risk score. Bold values represent the statistically significant.

For the second model, with age included, the estimated difference in log_10_ transformed total WMH volumes between the low and high DTC group was 0.133 but was no longer statistically significant (*p* = 0.07); however, the adjusted *R*^2^ value increased from 0.083 with no covariates to 0.413 for total WMH, with similar trends for all lobes, suggesting age significantly improved the model. While the difference in WMH in the frontal lobe remained significant (difference = 0.154, *p* = 0.03), the parietal and occipital lobes no longer differed between the low vs. high DTC group for counting.

Including the Framingham cardiovascular risk score as a covariate (model 3) resulted in an estimated difference in log_10_ transformed total WMH volumes of 0.163 (*p* = 0.03) between the low and high DTC group. The adjusted *R*^2^ value increased slightly to from 0.413 to 0.428 for total WMH. In this model, the frontal and parietal log_10_ transformed WMH volumes were greater in the high DTC group (difference = 0.174, *p* = 0.02 for frontal, difference = 0.213, *p* = 0.03 for parietal).

In the fourth model, which included age, cardiovascular risk, and MoCA as covariates, the estimated difference between the log_10_ transformed total WMH volumes of the low and high DTC groups was 0.150 but was not statistically significant (*p* = 0.053). For total WMH, the adjusted *R*^2^ value slightly decreased to 0.423 from 0.428 with the addition of MoCA. Frontal lobe WMH, however, remained significantly different between the high and low DTC groups (difference = 0.166, *p* = 0.03). Additional modeling with MDS-UPDRS III as a covariate did not substantially change the above results (see Supplementary material).

#### Fluency dual task cost

For all models, the difference in the log_10_ transformed WMH volumes were not significantly different between the low vs. high DTC with the exception of the occipital lobe using the model with no covariates (difference = 0.177, *p* = 0.04). Similar to counting DTC, adding age improved the model with the adjusted *R*^2^ increasing from 0.026 to 0.388 for total WMH burden with similar changes for all lobes; however, WMH burden was not significantly different between the low and high DTC groups for either the total or any of the lobes. The addition of the Framingham cardiovascular risk and MoCA, as well as additional modeling with MDS-UPDRS III (see Supplementary material), did not alter the results.

#### Serial 7s dual task cost

In the model with no covariates (Table 3), the log_10_ transformed WMH volume between low and high DTC groups was significantly different for total volume (difference = 0.181, *p* = 0.04) as well as in the frontal (difference = 0.180, *p* = 0.03) and occipital lobes (difference = 0.177, *p* = 0.04). After controlling for age (model 2), the estimated differences in log_10_ transformed total or lobar WMH volumes were no longer significant. The adjusted *R*^2^ value for total WMH burden increased from 0.040 from the first model to 0.400 for the second model; again; suggesting the addition of age significantly improved the model. Modeling with the Framingham cardiovascular risk and MoCA did not alter this result (Table 3), nor did the addition of MDS-UPDRS III as a covariate (Supplementary material).

## Discussion

The goal of this study was to assess the relationship between WMH burden and DTC across the spectrum of Lewy body related diseases. Baseline gait speed differed between the LBD groups, decreasing with increasing cognitive impairment (Table 1) and consistent with other studies (Mielke et al., 2012; Doi et al., 2014; Stegemöller et al., 2014). Similarly, WMH increased with increasing cognitive impairment. Under all conditions, the low DTC group was younger, had faster usual gait speed, was less cognitively impaired, and had lower WMH burden corresponding (Figure 1). Consistent with studies showing frontal brain areas are relevant for dual task walking performance (Kim and Fraser, 2022), dual task gait costs were associated with increased global WMH volume and most consistently with greater frontal WMH volume in the simplest counting backward task. We conclude that DTC may differ based on the secondary task and that WMH are associated with DTC. Age was a significant covariate. The associations were not statistically significant for the other dual task conditions after adjusting for covariates. The influence of different dual tasks on gait may differ between different patient populations (Smith et al., 2017; Raffegeau et al., 2019).

In a CU elderly population, increased volume of deep WMH was associated with slower walking speed under dual task conditions (Ghanavati et al., 2018). A study of patients with dementia showed white matter tract integrity was associated with lower speed under dual task conditions supporting an association between white matter changes and dual task gait performance (Hairu et al., 2021). Toda et al. (2019) concluded that higher WMH burden, measured with Scheltens visual rating scale, was associated with slower speed under dual task conditions in PD.

The overall model between median split DTC and log_10_ transformed WMH volume was statistically significant for the counting and serial 7s DTC conditions, but not for fluency DTC, without covariates (Table 3). Low or high DTC group membership may explain a statistically significant portion of the variance in the total WMH burden. It is not clear why results were only significant after age-adjustment for the counting DTC, but not the other tasks given that some studies suggests that DTC does not depend on the secondary task. One possibility is that there are differences in the impact of different DTCs in the population studied. Alternatively, participants may not have persisted in performing the secondary task. Adding age to the model significantly increased the adjusted *R*^2^ values for the whole model for all conditions, indicating age is an important factor that is associated with WMH and dual task gait (Table 3). The literature parallels this finding showing DTC increases and overall cognitive ability decreases, while WMH burden increases, with age (Murman, 2015; Sartor et al., 2017; Garnier-Crussard et al., 2020). Future studies should examine age-related imaging changes, such as brain atrophy and structural and functional connectivity.

Adding cardiovascular risk and MoCA did not significantly increase the adjusted *R*^2^ values (Table 3; Moroni et al., 2018). In a study of individuals with severe cerebral small vessel disease, in a younger population, single task and dual task gait performance was relatively preserved and showed little decline compared to healthy controls (Finsterwalder et al., 2019). In our sample WMH increased with cognitive impairment raising the possibility of a neurodegenerative contribution to white matter changes (Dadar et al., 2022). Although a positive correlation between cognitive function and gait speed under normal and dual task conditions has been shown (Chen et al., 2021), the lack of effect seen when MoCA is added to the model suggests that the relationship between DTC and WMH is not solely driven by global cognitive ability. In contrast, Ghanavati et al. (2018) found the relationship between deep WMH and dual task gait was not statistically significant after controlling for global cognition or executive function; however, the participants were cognitively normal community dwelling individuals.

One concern regarding the current study is data collection across multiple sites; however, this was mitigated with harmonized gait and imaging protocols. Furthermore, by using speed measures from electronic walkways (vs. stopwatch) and automated WMH volume measurements, variability and subjectivity was minimized. A major limitation of this study is sample size. Expanding the study to the rest of the COMPASS-ND cohort, which has stopwatch gait data for all participants could address this or examining other cohorts that include the spectrum of LBD (Montero-Odasso et al., 2020). Another limitation is that we did not examine the patients prior to and following taking medications, and hence cannot comment on the impact of medications on DTC and its associations. While many patients had a history of freezing of gait this did not interfere with gait performance, consistent with the patients being in an ON state (Camicioli et al., 1998). We provide the average freezing of gait score for descriptive purposes, but did not specifically examine differences in DTC between patients with or without freezing of gait.

The Lewy body group was treated as a continuous group, given that Lewy body pathology defines the population; but it is possible that other pathologies, particular Alzheimer pathology (Dadar et al., 2021), in addition to vascular pathology are present. Mixed pathology or misdiagnosis could influence the results.

Given sample size, associations between specific cognitive subdomains, dual task gait, and WHM were not examined. In a study of mild cognitive impairment, Alzheimer’s disease, and cognitively normal individuals, the visuospatial domain of the MoCA was independently associated with dual task gait measures (Ansai et al., 2017). Another study – of active cognitively normal elderly individuals – also found an association between dual task and the visuospatial/executive domains of the MoCA and Mini-Mental State Exam (Lima et al., 2015). The lack of change observed when MoCA was included in present analyses, could be due to a specific aspect of cognition driving the relationship rather than global cognition. A future direction could be to use MoCA domains or other domain-specific neuropsychological tests to determine which are related to DTC in the Lewy body spectrum and other neurodegenerative disorders with different cognitive profiles, given that stop-watch based DTCs will be available in COMPASS-ND (Montero-Odasso et al., 2020).

Future work could also include a more extensive analysis of dual task gait parameters. Dual task costs could be calculated for gait measures other than speed, like stride length or other specific gait domains, as they may show distinct relationships with specific regional brain changes (Murray et al., 2010; Lord et al., 2012; Pieruccini-Faria et al., 2021).

Some participants may have given up on the verbal tasks midway, as suggested by lower performance in the fluency and serial 7s tasks in the cognitively impaired groups. Such behaviors would not be adequately represented by measures that are averaged over the walking distance, and may account for the lack of statistically significant associations in these models. Additionally, the analysis did not control for the possibility of a cueing effect, whereby participants walk faster while completing a rhythmic task like counting.

## Conclusion

In this study, higher dual task gait cost with counting backward was associated with increased frontal WMH burden in participants across the spectrum of LBD, independent of age, vascular risk and MoCA score. However, the relationship appears to be strongly influenced by age. The impact of age-related neurodegeneration should be examined in future analyses and specific interventions, such as dual task training, should be examined to improve DTC. The study provides support for using the counting DTC as a practical task and motivates further examination of the neural substrates of DTC.

## Data availability statement

The datasets presented in this article are not readily available because data will become publicly available after the data acquisition phase of the COMPASS-ND study is completed and 1 year has passed. Requests to access the datasets should be directed to randi.pilon@ladydavis.ca.

## Ethics statement

The studies involving human participants were reviewed and approved by the University of Alberta Health Ethics Research Board. The patients/participants provided their written informed consent to participate in this study.

## Author contributions

IF drafted the manuscript with MG, KN, and RC. MG, KN, BS, MM, SB, ES, FP-F, and MM-O contributed to acquisition of data. IF, MG, FB, MD, SD, BS, MM, SB, ES, QA, and FP-F contributed to data analysis. RC provided the overall project oversight. All authors contributed to conceptualization of the project and reviewed the manuscript.
