# Supplementary material for: Dual-task gait and white matter hyperintensities in Lewy body diseases: An exploratory analysis
Source: Front Aging Neurosci. 2023 Apr 5;15:1088050. doi: 10.3389/fnagi.2023.1088050 (PMC10113527; doi:10.3389/fnagi.2023.1088050)
Supplement: Supplementary file 1 [file Table_1.pdf]

Supplementary Table: Modelling with UPDRS III

p-values are for the main effect of the DTC group (high vs low).							
		Counting DTC		Fluency DTC		Serial 7s DTC	
Log_{10} of:	covariates	Adj R-Sq	*p*-value	Adj R-Sq	*p*-value	Adj R-Sq	*p*-value
Total	none	0.08	**0.006**	0.03	0.08	0.04	**0.04**
	age	0.41	**0.07**	0.39	0.6	0.40	0.2
	age, MoCA	0.41	0.1	0.39	0.99	0.40	0.4
	age, FCRS	0.43	**0.03**	0.39	0.6	0.40	0.2
	age, CVR, MoCA	0.42	**0.05**	0.39	0.9	0.40	0.4
	age, log_{10}(UPDRS III)	0.47	**0.05**	0.44	0.7	0.44	0.3
	age, log_{10}(UPDRS III), MoCA	0.46	0.06	0.43	0.9	0.44	0.4
	age, log_{10} (UPDRS III), FCRS	0.47	**0.03**	0.43	0.7	0.44	0.3
	age, log_{10} (UPDRS III), FCRS, MoCA	0.46	**0.03**	0.43	0.8	0.43	0.3
Frontal	none	0.10	**0.003**	0.02	0.1	0.04	**0.03**
	age	0.41	**0.03**	0.37	0.8	0.39	0.1
	age, MoCA	0.41	**0.05**	0.37	1	0.39	0.3
	age, FCRS	0.41	**0.02**	0.37	0.7	0.38	0.2
	age, FCRS, MoCA	0.41	**0.03**	0.37	1	0.38	0.3
	age, log_{10} (UPDRS III)	0.45	**0.02**	0.41	0.8	0.42	0.2
	age, log_{10}(UPDRS III), MoCA	0.44	**0.03**	0.40	0.97	0.41	0.3
	age, log_{10} (UPDRS III), FCRS	0.44	**0.02**	0.40	0.8	0.41	0.2
	age, log_{10} (UPDRS III), FCRS, MoCA	0.44	**0.02**	0.39	1	0.40	0.3
Temporal	none	0.02	0.1	0.02	0.09	0.02	0.1
	age	0.31	0.6	0.31	0.6	0.32	0.3
	age, MoCA	0.30	0.7	0.31	0.7	0.31	0.4
	age, FCRS	0.35	0.3	0.34	0.5	0.35	0.3
	age, FCRS, MoCA	0.34	0.3	0.33	0.5	0.34	0.3
	age, log_{10} (UPDRS III)	0.38	0.6	0.37	0.6	0.38	0.5
	age, log_{10}(UPDRS III), MoCA	0.37	0.5	0.37	0.5	0.37	0.4
	age, log_{10} (UPDRS III), FCRS	0.40	0.3	0.40	0.6	0.40	0.4
	age, log_{10} (UPDRS III), FCRS,MoCA	0.40	0.2	0.39	0.5	0.40	0.3
Parietal	none	0.08	**0.008**	0.03	0.07	0.03	0.06
	age	0.41	0.08	0.38	0.5	0.39	0.2
	age, MoCA	0.41	0.2	0.40	0.9	0.40	0.5
	age, FCRS	0.43	**0.03**	0.40	0.5	0.40	0.3
	age, FCRS, MoCA	0.43	0.07	0.40	0.8	0.40	0.5
	age, log_{10} (UPDRS III)	0.49	**0.05**	0.46	0.6	0.47	0.4
	age, log_{10}(UPDRS III), MoCA	0.48	0.07	0.46	0.8	0.46	0.6
	age, log_{10} (UPDRS III), FCRS	0.49	**0.03**	0.46	0.6	0.46	0.4
	age, log_{10} (UPDRS III), FCRS, MoCA	0.49	**0.04**	0.46	0.8	0.46	0.5

Occipital	none	0.04	**0.04**	0.04	**0.04**	0.01	0.2
	age	0.25	0.2	0.25	0.2	0.24	0.5
	age, MoCA	0.29	0.5	0.28	0.6	0.28	1
	age, FCRS	0.27	0.1	0.26	0.2	0.25	0.5
	age, FCRS, MoCA	0.29	0.3	0.28	0.6	0.28	1
	age, log_{10} (UPDRS III)	0.28	0.2	0.28	0.3	0.27	0.7
	age, log_{10}(UPDRS III), MoCA	0.30	0.4	0.29	0.6	0.29	0.9
	age, log_{10} (UPDRS III), FCRS	0.29	0.1	0.28	0.3	0.27	0.6
	age, log_{10} (UPDRS III), FCRS, MoCA	0.30	0.3	0.29	0.6	0.28	1
